# François Pourfour du Petit (1664–1741): a pioneer in experimental medicine

**DOI:** 10.1007/s00701-026-06820-8

**Published:** 2026-03-12

**Authors:** Sefa Öztürk, Abuzer Güngör, Mazlum Işik, Wolfgang J. Weninger, Uğur Türe

**Affiliations:** 1 Department of Neurosurgery, Istanbul University Istanbul Faculty of Medicine, Istanbul, Türkiye; 2 Department of Neurosurgery, Yeditepe University School of Medicine, Istanbul, Türkiye; 3 Department of Neurosurgery, İstinye University School of Medicine, Istanbul, Türkiye; 4 Department of Neurosurgery, Dortyol State Hospital, Hatay, Türkiye; 5 Division of Anatomy and Medical Imaging Cluster, Medical University of Vienna, Vienna, Austria

**Keywords:** Cataract surgery, Decussation of the pyramidal tract, Experimental medicine, Intercostal nerves, Petit syndrome

## Abstract

François Pourfour du Petit (1664–1741) is one of the most versatile scientists in medical history. Trained as a surgeon, chemist, ophthalmologist, and botanist, Petit was an intellectual who combined science with observation and experimentation. Through meticulous examination of the complex structure of the human nervous system, Petit provided important experimental observations on the decussation of the pyramidal tract and offered influential insights into the origin of the sympathetic nervous system. These discoveries directly influenced both the understanding of neurological injuries and the development of surgical interventions. His medical education began in 1687 as a student of Pierre Chirac at the Faculty of Medicine in Montpellier, culminating in his receiving the title of doctor in 1690. In Paris, at the Jardin Royal des Plantes, he studied under leading scientists of the time, including M. Duverney, M. de Tournefort, and M. Lémery, performing dissections and surgeries. While serving in Louis XIV's army, he observed neurological injuries in patients in military hospitals, providing revolutionary insights into the relationship between the nervous system and motor functions. Petit's meticulous measurements and experimental approach influenced not only brain anatomy but also eye anatomy and cataract surgery. While working as an ophthalmologist in Paris, he successfully performed cataract surgery on a woman in Fresnes in 1726, restoring her vision. Pourfour du Petit died after undergoing surgery for a long-standing recurrence of a hernia. He left behind not only his observations but also a methodological legacy aimed at understanding the structure of the human brain. His work remains a guiding light in the literature of neuroanatomy and brain surgery, ensuring that he is remembered as one of the pioneers of modern medicine.

## Introduction

**Fig. 1:**
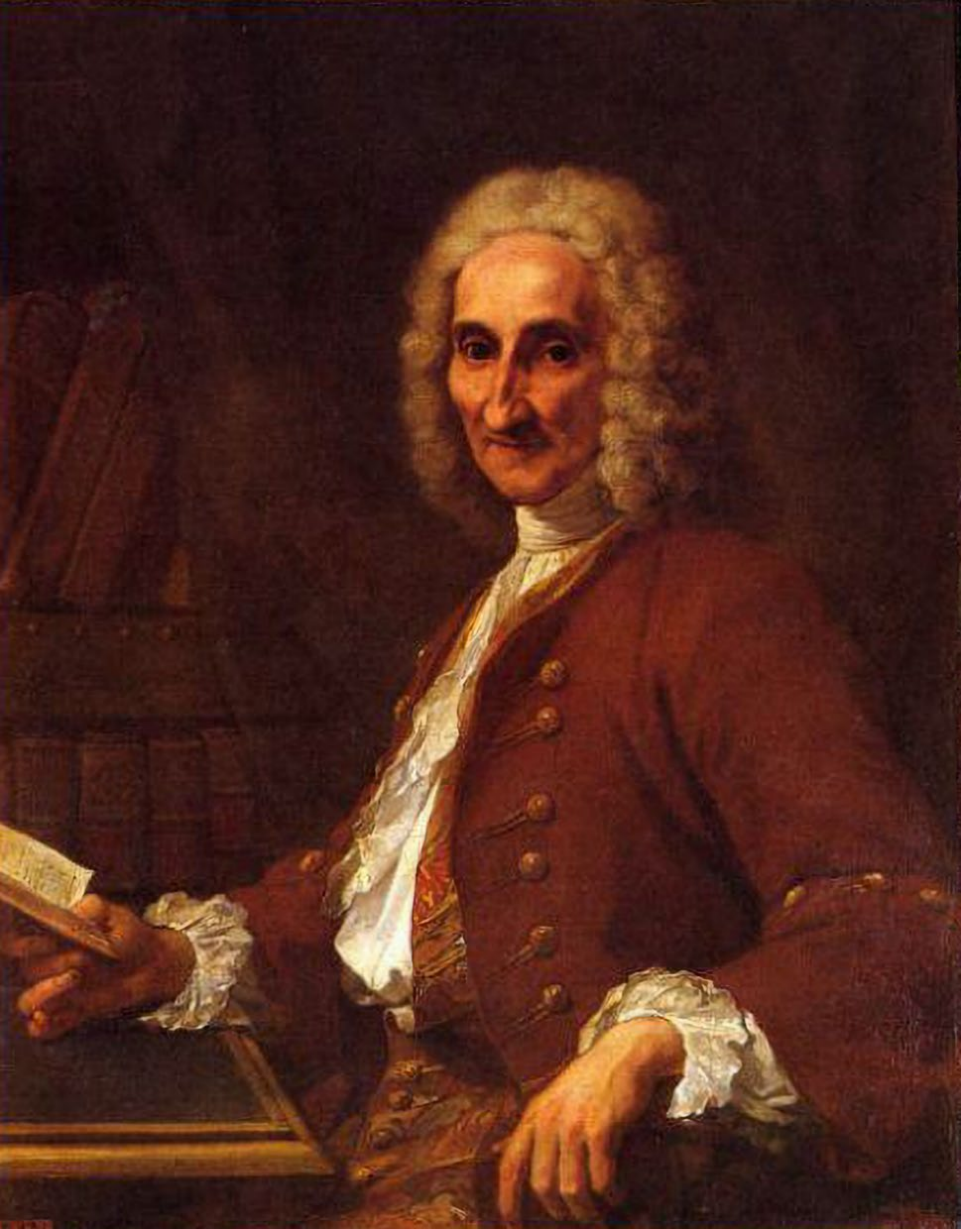
François Pourfour du Petit (1664–1741), French anatomist and physician (Public domain image. Source: Wikimedia Commons.)

The Enlightenment era is widely regarded as marking a transformative phase in the history of Western medicine and science, with a shift towards a greater emphasis on empirical observation and experimentation in place of traditional doctrine. In this intellectual environment, anatomists, physicians and surgeons pursued more systematic and evidence-based practices than in previous centuries. François Pourfour du Petit (1664–1741) is a perfect example of this transition, bridging the gap between anatomical theory and experimental physiology (Fig. 1). Notable strides were made in ocular biometry and surgical methods, but it is chiefly his research on the decussation of the pyramidal tract and the sympathetic nervous system that elevates his legacy in the history of neuroscience [7, 26, 32, 38].

Despite his important contributions, François Pourfour du Petit’s work was often overshadowed or confused with that of his contemporaries. He placed little value on style or literary polish in his writings, considering them unnecessary for scientific work. Instead, he focused strictly on facts: he observed carefully, recorded with precision, and avoided unnecessary debate. He presented his findings clearly and without ornamentation, yet always with attention to detail. His commitment to accuracy was absolute, and he rejected even minor embellishments that might distort the truth [14, 41].

His life, which began in Paris and involved extensive military medical service, placed him in direct contact with battlefield injuries, allowing him to gather substantial clinical and anatomical observations. The systematic approach he adopted (combining post-mortem dissections, live animal experiments, and clinical case analyses) epitomizes the experimental paradigm that would later become a cornerstone of modern medical research [4, 26]. This article examines the historical-scientific context of Pourfour du Petit’s era, traces his education and career trajectory, and surveys his principal discoveries in neuroanatomy, neurophysiology, and ophthalmology. Through contemporary accounts, later commentaries, and his own letters, this study shows that he was both a pioneer of scientific methods and a thinker whose ideas strongly influenced later developments [26, 32].

## François Pourfour du Petit’s education and early career

François Pourfour du Petit was born in Paris in 1664 and lost both parents at an early age. He received his preliminary education at the Collège de Beauvais, where courses in natural history, chemistry, and anatomy left a lasting impression. The connections between these disciplines and medicine drew him toward clinical practice and engagement with established physicians [14, 30, 41].

**Fig. 2:**
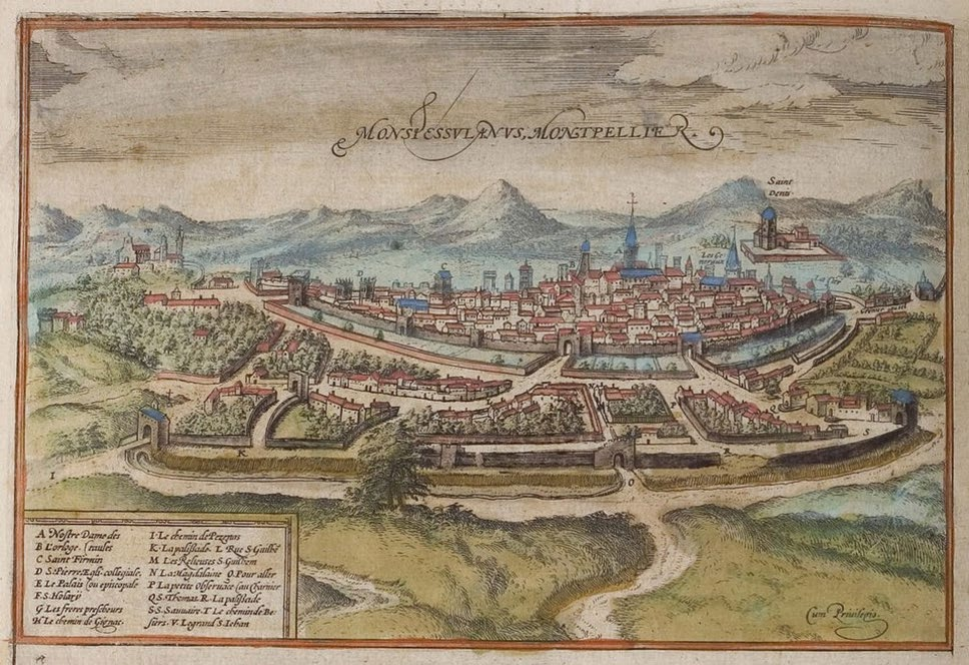
A 1572 view of Montpellier from Civitates Orbis Terrarum, Volume I, by Georg Braun and Frans Hogenberg, depicting the city where Pourfour du Petit pursued part of his medical education (Public domain image. Source: Wikimedia Commons.)

In the late 1680 s, Petit enrolled at the University of Montpellier (Fig. 2), which was gaining renowned for its progressive medical curriculum emphasizing direct anatomical dissection and clinical observation. Under the guidance of Pierre Chirac, he explored diverse medical fields while also attending chemistry courses. In 1690, he obtained his medical degree and returned to Paris at the age of 26. There, he attended public lectures at the Jardin du Roi by Joseph Guichard Duverney [33] (anatomy) and Joseph Piton de Tournefort [15] (botany), as well as private chemistry instruction from Nicolas Lémery [27]. These experiences allowed Petit to consolidate a strong foundation in anatomy, botany, and experimental science while gaining the respect and mentorship of the leading scholars in Paris [10, 39].

Petit’s practical approach led him to gain direct experience in surgery and clinical care. For six months, he dressed wounds at La Charité Hospital, developing both technical skills and patient care abilities. Between 1691 and 1692, during France’s military campaigns, he worked in field hospitals in Flanders, including Mons, Namur, and Dinant [14, 41]. After completing his early military and hospital work, Pourfour du Petit continued serving as a physician and surgeon in the French army through the mid-1690 s into the early 1700 s, particularly during the War of the Spanish Succession (1701–1714). Exposure to battlefield injuries-including penetrating head wounds and ocular trauma-provided invaluable material for his research. Notably, the occurrence of paralysis contralateral to the site of brain injury in soldiers with acute head trauma sparked his intense scientific curiosity. By systematically observing and comparing data from dissections and patient outcomes, Petit established increasingly precise correlations between localized brain lesions and the resulting motor deficits or ocular changes [16, 30].

**Fig. 3:**
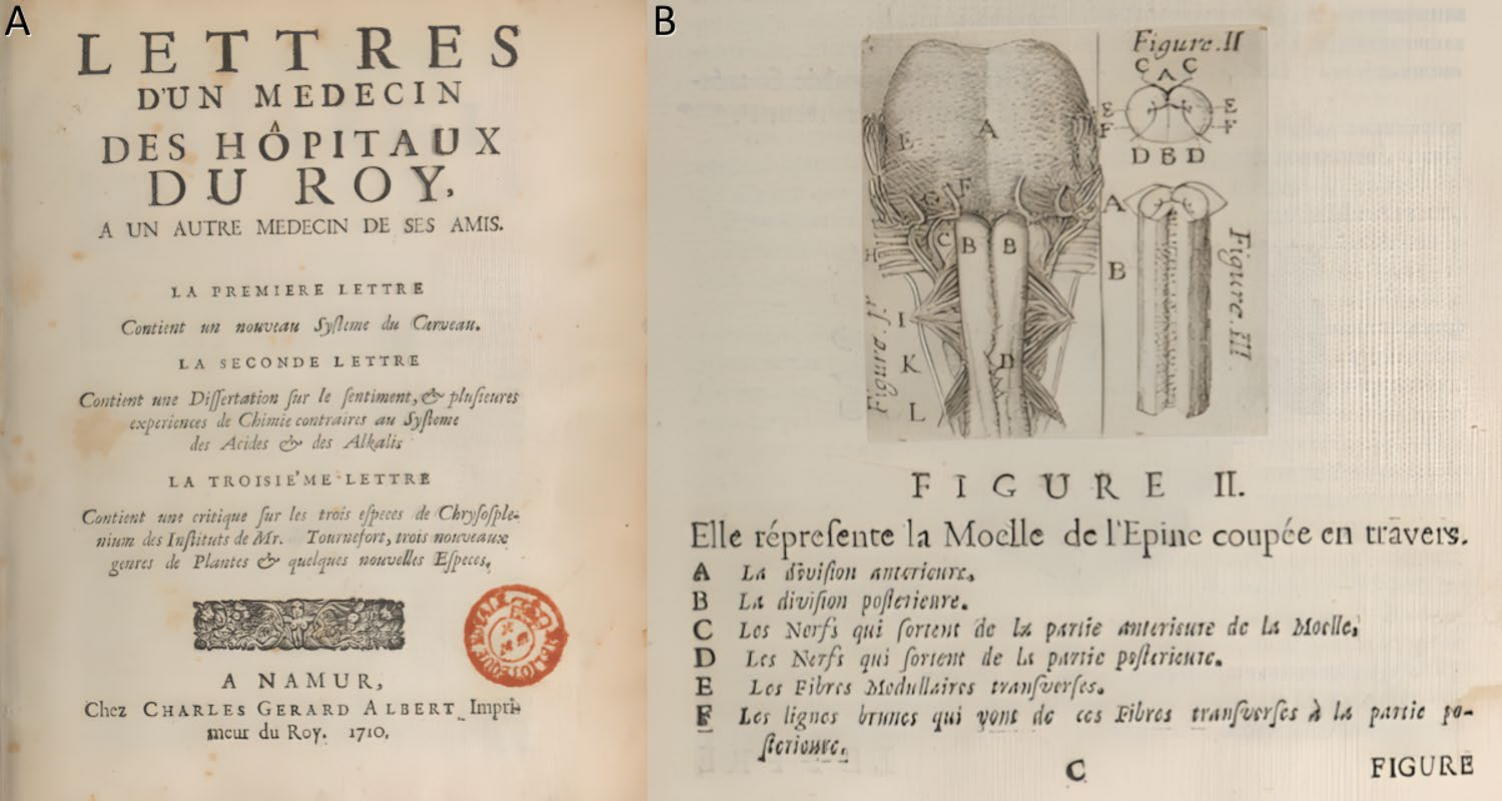
**a** Title page of Lettres d’un Médecin des Hôpitaux du Roy, à un autre Médecin de ses amis, Pourfour du Petit’s influential collection of medical letters [15]. **b** Original illustration from the same work depicting his first description of the decussatio pyramidalis [15]

In 1710, Petit published Lettres d’un Médecin des Hôpitaux du Roy, à un autre Médecin de ses amis [16, 31] (Fig. 3), in which he elaborated on the concept of “contre-coup” in head injuries and early ideas about motor fiber decussation [19]. This work garnered attention within academic circles, especially among researchers of the nervous system. Following years of travel and medical service, Pourfour du Petit settled in Paris in 1713 at the age of 49, shortly after the signing of the Treaty of Utrecht. In 1717, he married and the couple had four children, three of whom died at a young age. His eldest son, Étienne Pourfour du Petit, followed in his father's footsteps and pursued a career in medicine, eventually serving as dean of the Faculty of Medicine in Paris from 1781 to 1782 [14].

In 1722, Petit was elected an associate member of the Académie Royale des Sciences and, in 1725, he retired from active clinical service to dedicate himself fully to experimental and anatomical research [41]. Petit established himself in Paris as an ophthalmologist and performed several cataract operations. However, rather than seeking wealthy clientele, he dedicated himself to restoring sight to the poor. In 1726, according to De Mairan [14], he successfully restored the vision of a 60-year-old woman at the residence of the chancellor in Fresnes. As he aged, Petit gradually ceased performing surgeries, devoting himself instead to anatomical investigation. His primary concern was not personal wealth but empirical observation and medical experience. He focused intensively on the anatomy of the eye and the refinement of new cataract surgery techniques. Known for his rigorous fidelity to observation, Petit rarely engaged in theoretical debate, preferring to record and report findings in the order they were observed, with notable precision and honesty.

Petit’s position within the Academy consolidated his reputation further. He collaborated with leading anatomists and physicians examining the brain, spinal cord, and ocular system. Through both independent and collaborative efforts, he refined the arguments set forth in his earlier letters and developed innovative anatomical techniques to study human and animal specimens. Throughout his career, Petit remained committed to empirical observation, methodological rigor, and the ethical practice of medicine, leaving a lasting mark on neuroscience and clinical anatomy [7, 26, 30, 41].

## Key contributions to neuroanatomy, neurophysiology and ophthalmology

### On the pyramidal decussation: petit’s experimental observations

Although the phenomenon of contralateral hemiplegia after brain injury had been clinically recognized well before the eighteenth century, the anatomical explanation remained speculative. Ancient physicians such as Cassius [37] and Aretaeus [3, 40] had proposed that nerve pathways crossed from one hemisphere to the other to account for the lateralization of motor deficits, but these assertions were grounded more in philosophical reasoning than empirical observation [14]. By the early modern period, dominant theories still relied on metaphysical constructs, such as “animal spirits” flowing through cerebral ventricles, or vague notions of *contre-coup* injuries manifesting on the side opposite the trauma. In this context, François Pourfour du Petit undertook a systematic investigation to anatomically and physiologically explain this clinical pattern. His approach-integrating battlefield clinical observations, autopsy findings, and experimental animal studies-allowed him to provide substantial experimental support for a long-standing hypothesis. Although the first anatomical description of the pyramidal decussation in the upper medulla oblongata was reported by Domenico Mistichelli in 1709 [12, 29, 42], Pourfour du Petit provided important experimental observations on contralateral motor deficits and the crossing of descending motor fibers in his 1710 publication. In this work, composed of three letters and printed in a limited number of copies, he discussed in detail the anatomical implications of motor fiber crossing-particularly in the second letter-while also addressing contemporary fluid-based theories of nerve transmission that were prevalent at the time [6, 7, 41] (Table 1).

**Table 1:** Scientific contributions of François Pourfour du Petit (1664–1741) to neuroanatomy, neurophysiology, and ophthalmology

Year(s)	Contribution/Discovery	Methodology/Context	Significance
1710	Experimental demonstration of pyramidal decussation in the medulla oblongata​​​	Clinical observations on wounded soldiers, cadaveric dissections, and animal experiments	Established anatomical basis of contralateral paralysis (hemiplegia), foundational for modern neurology
1710–1729	Clarification of sympathetic nervous system origins in thoracic and lumbar spinal segments​	Animal vivisection, systematic anatomical dissections	Corrected the misconception of cranial origin (intercostal nerve theory), reshaping autonomic nervous system knowledge
1710	Early contributions to cerebellar physiology	An experiment in which partial removal of a dog’s cerebellum caused the animal’s body to bend toward one side in a rigid, arched posture	Questioned the cerebellum’s role in sensory processing, suggesting instead its involvement in motor coordination
1712–1726	Development of ocular biometric techniques (lens and corneal measurements)	Innovative methodologies including frozen ocular sections and immersion technique	Pioneered quantitative methods in ocular anatomy, forming the basis of modern ophthalmic biometrics
1710–1726	Publication of “Dissertation sur une nouvelle méthode de faire l’opération de la cataracte,” which focused on refinements in cataract surgery and proposed extracapsular extraction techniques	Surgical practice and innovation based on clinical experience	Improved surgical techniques, significantly influenced ophthalmic surgical practices
1722	Elected member of the Académie Royale des Sciences​	Recognition of his rigorous experimental and anatomical methods	Validated and disseminated his experimental methods, enhancing the credibility and influence of subsequent research
1726–1729	Description of “Pourfour du Petit syndrome”: ocular changes due to cervical sympathetic chain manipulation​​	Surgical experiments on cervical sympathetic trunk in animal models	Early recognition of sympathetic ocular syndromes; precursor to understanding Horner syndrome

Petit’s initial clinical evidence was drawn from wounded soldiers treated during military campaigns [16]. Across five separate cases, he consistently observed the same pattern: cranial trauma on one side, followed by severe headache and motor deficits-usually hemiplegia-on the contralateral side, and, in some cases, death. These injuries included sabre wounds, gunshot wounds, and blunt trauma. One notable case involved a soldier who sustained a sabre cut to the right lower eyelid and presented ten days later with severe orbital inflammation, exophthalmos, and complete paralysis of the left upper extremity. Phlebotomy was done, after which the soldier’s headaches diminished, and motor function gradually returned. The reproducibility of contralateral motor impairment across varying mechanisms of injury prompted Petit to suspect a structural crossing of motor pathways within the brainstem.

One particularly illustrative case involved a high-ranking officer who sustained a localized sabre wound just beneath his right lower eyelid, near the anterior branch of the fifth cranial nerve. Although the superficial wound healed within two days, the patient soon developed a severe headache and progressive weakness in his left arm, which became nearly immobile. Despite continued treatment, his condition deteriorated, and he died within three months. This case, consistent with Petit's other battlefield observations of contralateral motor deficits after unilateral cranial injuries, became central to his investigation. During autopsy, Petit found no apparent damage at the external injury site. However, while removing the brain from the skull base, he discovered that the dura mater was abnormally adherent to the region above the orbit and to the origins of the extraocular muscles, a finding that immediately suggested localized inflammation. As the adherent dura was separated, a tear occurred in the pia mater, creating an opening through which a large amount of thick, greenish-white, porridge-like pus escaped from the anterior aspect of the brain near the optic nerve. Initially, Petit suspected that the purulent material might have originated from the right ventricle, given the right-sided injury, but once the brain was fully detached and the right ventricle was opened-after excising part of the middle and inferior lobes-its clear cerebrospinal fluid confirmed that it was not the source. Exploration of the drainage tract revealed a cavity approximately two fingers in width and depth, corresponding to an extensive abscess located within the fibrous and medullary tissues overlying the so-called external process, in the region covering the outer canalicular structures (canalicular structures (corps cannelés): a term used in that period for the corpus striatum) of the left hemisphere, all of which were found to be completely destroyed. After examining similar cases, Petit consistently noted this contralateral pattern of internal inflammation, strengthening his argument that motor pathways cross within the brainstem-a foundational insight that he formally articulated in his 1710 treatise [10, 16, 41].

To reinforce his findings, Petit conducted a series of experiments on dogs. In one experiment, he trephined the left parietal bone and introduced a transverse incision into the cerebral cortex, cutting from right to left and posterior to anterior. Hours later, the animal lost motor function in its right limbs, while the left side remained unaffected. By the next day, the dog showed partial motor recovery and was able to stand intermittently using only the weakened right forelimb. It died 76 h later. Autopsy revealed significant fluid accumulation within the left hemisphere. Across multiple animals, similar patterns of contralateral motor impairment were consistently reproduced. These results provided additional physiological support for the concept of decussation at the level of the medulla oblongata [19, 30].

Building on his experimental results, Petit proposed a detailed anatomical model of motor decussation. He described how cortical gray matter projects motor signals via descending medullary fibers, which traverse structures such as the corpus callosum and converge in the lower brainstem. At the medulla, these fibers bundle into the pyramids, where they divide into two or three fascicles and interweave in a decussating pattern. Fibers originating from the right hemisphere cross to the left side of the spinal cord and vice versa. This anatomical crossing-now recognized as the pyramidal decussation- offered a coherent anatomical explanation for contralateral motor deficits following unilateral brain injury [10, 30].

Petit’s work was well received by his peers and earned him election to the Académie Royale des Sciences in 1722 [10, 14]. His demonstration that hemiplegia after focal brain trauma was not the result of fluid transposition or vague commotional effects but was due to the anatomically fixed decussation of motor fibers was transformative. His work strengthened earlier hypotheses by integrating clinical, pathological, and experimental observations into a more structured anatomical framework. This contribution would not only influence his contemporaries but would also be cited as a foundational discovery in the development of modern neuroanatomy.

### Historical evolution and pourfour du petit’s contribution to the anatomy of the sympathetic systems

The intercostal nerve (*The term “intercostal nerve” in ancient and early-modern anatomical texts does not correspond to the modern intercostal nerves. Historically, “intercostal nerve” referred to the sympathetic trunk, particularly the cervical and upper thoracic sympathetic chain* [7]) has been recognized since antiquity, having been named by both Hippocrates [36] and Galen [21]. Galen noted the difficulty of visualizing this nerve because it could easily be mistaken for the carotid artery, yet he maintained that it did not arise from the fifth and sixth cranial pairs but rather from the union of the third and fourth pairs (The “third,” “fourth,” “fifth,” and “sixth” pairs mentioned here do not correspond to the modern cranial nerve nomenclature but instead refer to the third through sixth pairs of Galen’s seven-nerve classification. Within this historical framework, the third and fourth pairs together encompass the structures corresponding to the modern trigeminal nerve (V), whereas the fifth pair roughly aligns with the facial–vestibulocochlear complex (VII–VIII), and the sixth pair corresponds to the glossopharyngeal–vagus–accessory complex (IX–XI) Table 2). In 1516, the Italian physician and philosopher Alessandro Achillini (1463–1512) published De Humani Corporis Anatomia [2], in which he proposed that the intercostal nerve was connected to the fifth and sixth cranial pairs(facial–vestibulocochlear complex and glossopharyngeal–vagus–accessory complex). A century later, Lancisi’s 1714 edition of Bartholomeo Eustachi’s anatomical plates [18] showed the intercostal nerve as forming an anastomosis with the sixth cranial pair (glossopharyngeal–vagus–accessory complex (IX–XI), reinforcing the view that many physicians subsequently adopted.

**Table 2:** Historical comparison of cranial nerve classifications in Galen [20], Thomas Willis [43], Von Soemmerring [41], and the modern anatomical system

Current Nerve Name	Galen Classification	Willis Classification	Von Soemmerring Classification
Olfactory	Not mentioned	1 st pair	I
Optic	1 st pair	2nd pair	II
Oculomotor	2nd pair	3rd pair	III
Trochlear	Not mentioned	4th pair	IV
Trigeminal	(3rd pair) Sensory root (4th pair) Motor root	5th pair	V
Abducens	Not mentioned	6th pair	VI
Facial	5th pair	7th pair	VII
Vestibulocochlear			VIII
Glossopharyngeal	6th pair	8th pair	IX
Vagus			X
Accessory			XI
Hypoglossal	7th pair	9th pair	XII
Suboccipital Nerve	-	10th pair	-

In the seventeenth century, Thomas Willis offered a more elaborate account. He described the intercostal nerve as being formed from two branches of the fifth and one branch of the sixth cranial pair (Willis’s cranial nerve classification, widely used in the late seventeenth and early eighteenth centuries, represents a transitional stage in neuroanatomical knowledge. In this system, several nerves were grouped differently from their modern counterparts, and the suboccipital nerve was even included among the cranial nerves. Notably, however, Willis’s fifth and sixth cranial pairs correspond closely to the modern trigeminal (V) and abducens (VI) nerves, reflecting an early convergence with contemporary anatomical concepts. Although superseded by Sömmerring’s [44] later scheme, Willis’s taxonomy remained influential for decades). This composite nerve, he argued, exited the skull through the same canal as the carotid artery and reached the olivary bodies. According to his schema, the fifth cranial nerve (trigeminal) divided into three branches: one supplying the levator palpebrae superioris, one to the upper jaw, and one to the lower jaw. The branch to the levator palpebrae, he maintained, received a contribution from the intercostal nerve (the structure corresponding to the sympathetic chain in modern anatomy). Furthermore, the sixth cranial nerve (Abducens), which innervates the lateral rectus muscle, issued a communicating branch that united with the fifth cranial nerve, thereby reinforcing the intercostal connection [46].

Raymond Vieussens (1635–1715), though broadly agreeing with Willis on the involvement of the fifth (trigeminal) and sixth (Abducens) cranial nerves, diverged on the issue of origin. He accepted that the intercostal nerve received a substantial contribution from the large anterior root of the fifth cranial nerve, but he insisted that such a communication could not be regarded as the nerve’s true source. This distinction sustained the ambiguity over the anatomical nature of the intercostal nerve [43]. It was precisely this unresolved conflict that later prompted François Pourfour du Petit to revisit the problem with renewed dissectional rigor and experimental evidence [45].

François Pourfour du Petit addressed this lacuna with meticulous dissection and experimental physiology. In his 1727 thesis, he challenged the dominant cranial-origin paradigm, asserting instead that the intercostal nerve (the structure corresponding to the sympathetic chain in modern anatomy) arises from the spinal cord and ascends toward the skull, contributing fibers to a complex carotid–sphenoidal plexus. He described a network of neural strands encircling the carotid that coalesce at the sellar region, with prominent connections to the anterior root of the trigeminal nerve and subsidiary links to the abducens. Crucially, Pourfour argued that many structures previously taken for the nerve’s origin were in fact peripheral branches or plexiform expansions-an observation with immediate bearing on both anatomical nomenclature and surgical exposure [1, 10, 19, 45].

Beyond static anatomy, Pourfour’s functional experiments recontextualized the sympathetic trunk. He proposed that the great sympathetic is fundamentally a spinal-derived system with predominant motor (vasomotor) functions rather than a purely sensory cranial extension. His observation that cervical cord transection in canines abolished certain pain responses anticipated later demonstrations of autonomic and vasomotor pathways-most notably those formalized by Claude Bernard (1813–1878)-and influenced subsequent approaches to autonomic neurophysiology and neurosurgical strategy. Collectively, Pourfour du Petit’s integrative dissectional and experimental work decisively reframed the craniospinal nerve relationships with enduring relevance to neurosurgery and neuroanatomy [7, 41].

### The emergence of Pourfour du Petit syndrome

François Pourfour du Petit’s experimental work on the cervical sympathetic chain laid the foundation for what would later be recognized as Pourfour du Petit syndrome-a rare neuro-ophthalmological condition characterized by mydriasis, eyelid retraction, mild exophthalmos, facial pallor, vasoconstriction, and sometimes hyperhidrosis. While investigating whether the intercostal nerves transmitted “animal spirits” to the eye, Petit performed pioneering sectioning experiments on the superior cervical sympathetic trunk. He noted consistent ocular and autonomic changes following these dissections, thus providing one of the earliest functional anatomical demonstrations of sympathetic innervation to the eye. His observations, published in 1726 and 1727, not only clarified the sympathetic origin of pupillary dilation but also linked the dilator pupillae muscle directly to the ciliary nerves and the broader sympathetic chain-well before the autonomic nervous system was fully conceptualized [1, 25].

During a series of experiments conducted in early 1712, Pourfour du Petit investigated the function of the cervical sympathetic chain by surgically severing the major sympathetic nerve connecting the superior and inferior cervical ganglia in dogs. His aim was to distinguish the specific ocular and autonomic effects of sympathetic lesions from those caused by damage to adjacent cranial nerves, such as the eighth pairs of cranial nerves (glossopharyngeal–vagus–accessory complex) which shared the same nerve sheath. In these trials, Petit consistently observed that unilateral or bilateral sectioning of the sympathetic nerve led to progressive ocular changes, including dullness of the eye, enophthalmos, miosis, lacrimation, and eventually blindness. In one experiment, cutting the right intercostal nerve resulted in ipsilateral loss of ocular brightness within 30 min; three days later, the eye became sunken and contracted. When the contralateral nerve was cut weeks later, similar degeneration occurred in the other eye. In his final experiment, bilateral sympathectomy caused the dog’s eyes to become so inflamed, sunken, and watery that it nearly lost vision. Petit interpreted these findings as clear evidence that the sympathetic nerves were responsible for transmitting “animal spirits” to the eyes-a notion that anticipated modern understandings of sympathetic ophthalmic function [1, 14].

Though largely forgotten for several decades, Petit’s findings gained renewed interest in the late 18th and early nineteenth centuries. Petri Paulli Molinelli reaffirmed Petit’s early descriptions in his 1755 article published in *Commentarii de Bononiensi Scientiarum et Artium Instituto atque Academia* [11], and the syndrome was further substantiated by Jean-Louis Brachet (1823) [9] and John Reid (1839) [35], all of whom focused on ocular manifestations after sympathetic lesions. These authors delineated two distinct sources of ocular innervation: one arising from the brain via the cranial nerves (optic, oculomotor, trochlear, and abducens), and the other from the sympathetic ganglionic system. Brachet, in particular, emphasized that pupillary dilation was under sympathetic control and that lesions to the cervical sympathetic chain led to its failure. Petit’s early anatomical and physiological insights thus anticipated the modern understanding of sympathetic ophthalmology and contributed directly to the definition of the syndrome that now bears his name.

### The cerebellum: early observations

Although Pourfour du Petit is primarily known for his work on the sympathetic nervous system, he also made remarkably early contributions to cerebellar physiology. In his 1710 letters, he described an experiment in which the partial removal of a dog’s cerebellum caused the animal’s body to bend toward one side in a rigid, arched posture. This observation led Petit to question the cerebellum’s role in sensory processing, suggesting instead its involvement in motor coordination [16, 30].

Over a century later, Magendie and Flourens [20, 41] expanded on these ideas. They demonstrated that lesions to the cerebellar peduncles in animals caused abnormal movements, especially axial rotation and postural instability. Although Magendie initially claimed priority in describing these phenomena, later scholars such as Henry Milne-Edwards pointed out that Petit had documented similar effects long before [28]. Most later researchers acknowledged Petit’s priority, recognizing his foundational insights [10, 41].

Additionally, some of Petit’s descriptions bear striking similarities to Babinski’s 20th-century concept of cerebellar asynergia. For instance, Petit described a patient with continuous involuntary movements despite normal strength and sensation, and also noted that dogs with cerebellar lesions could not maintain balance, showing the same arching of the back that Babinski would later characterize [5]. Though brief, Petit’s observations anticipated key principles of cerebellar dysfunction, bridging early experimental neurology with modern neurophysiology.

### Ocular biometry and ophthalmological innovations

#### Foundations of ocular biometry

Although Pourfour du Petit is most widely recognized for his contributions to neuroanatomy, his studies of ocular biometry are no less significant. During a period when the dimensions and optical properties of the eye were often discussed in purely theoretical terms, he pioneered practical measurement techniques for ocular structures. He measured parameters such as the thickness of the crystalline lens, the diameter of the eye, and changes in these structures with aging [1, 32].

One approach was to prepare frozen sections of animal and human eyes to overcome refraction artifacts and measure the lens more reliably. He also employed immersion techniques, suspending the eye in fluids to neutralize the cornea’s refractive power. These experimental setups, though rudimentary by modern standards, represented a methodologically advanced approach for the eighteenth century and allowed for more precise quantitative data [22, 41].

#### Advances in cataract surgery

Cataract surgery during Pourfour du Petit’s time most commonly involved the “couching” technique, in which the opaque lens was pushed to the bottom of the vitreous cavity with a needle, effectively removing it from the visual axis. This approach, however, carried significant risk of complications, including retinal detachment or severe inflammatory responses. Observing these issues, Pourfour du Petit investigated and advocated for more refined lens extraction methods, a stance that anticipated later developments by Jacques Daviel (1696–1762), who is credited with popularizing extracapsular cataract extraction [13, 17].

**Fig. 4:**
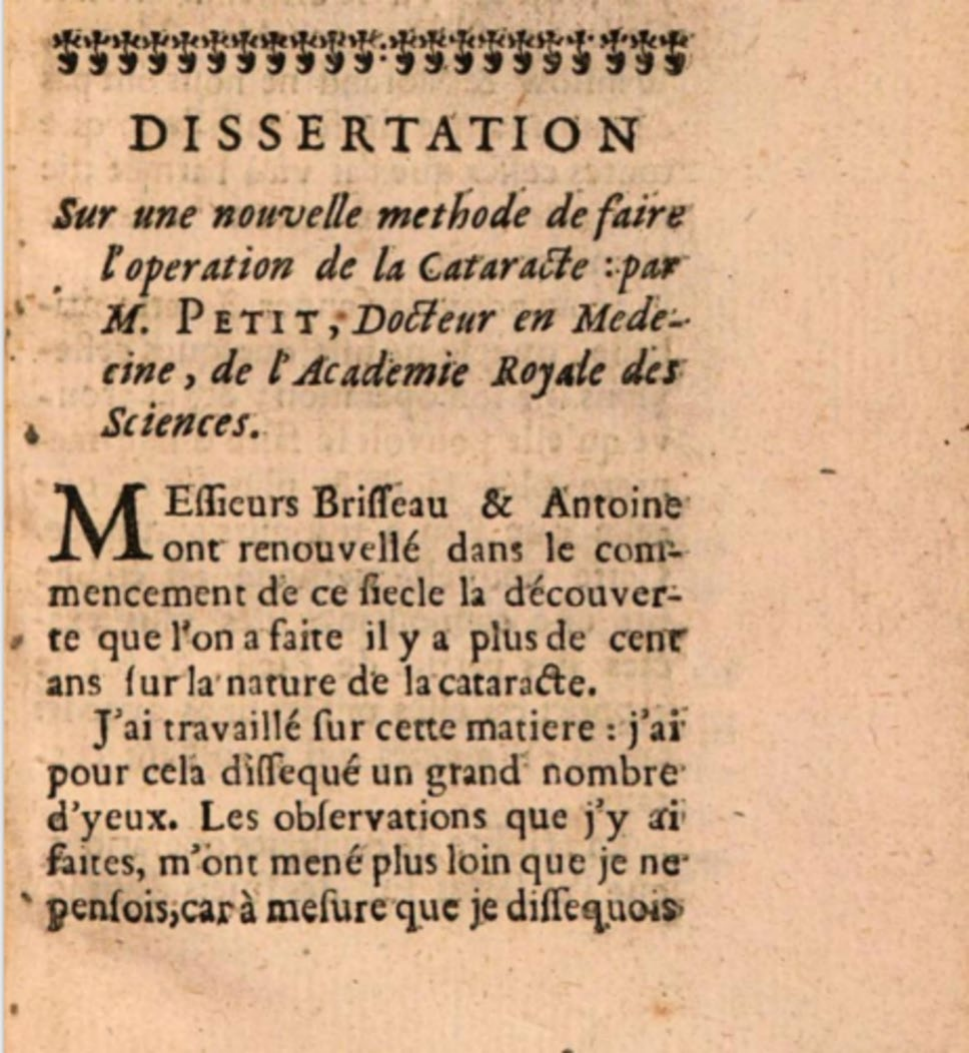
Title page of *Dissertation sur une nouvelle méthode de faire l'opération de la cataracte* by François Pourfour du Petit (Paris, 1726) [14]

**Fig. 5:**
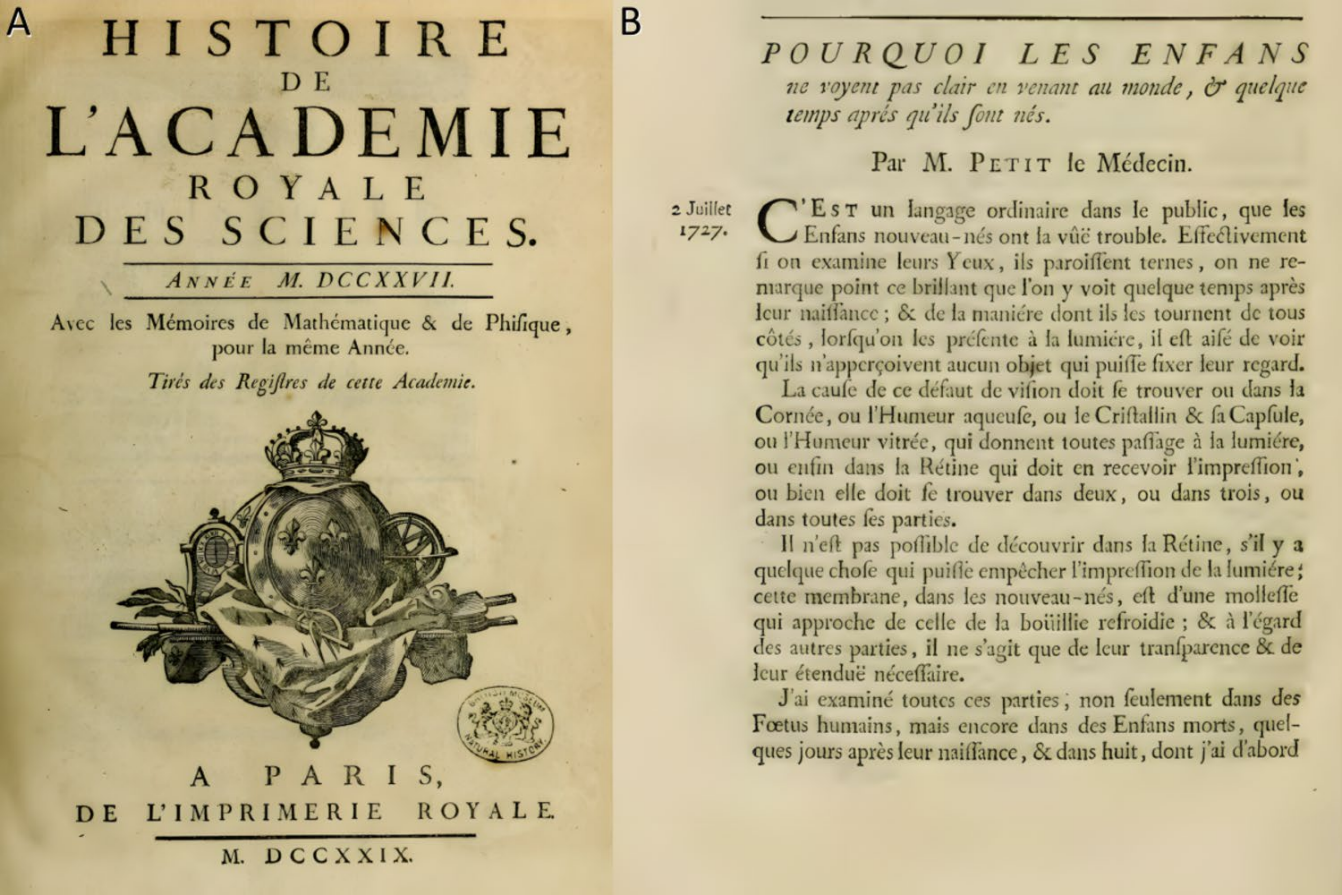
**a** Title page of Histoire de l’Académie Royale des Sciences for the year 1727 (published in 1729), featuring the official emblem of the Royal Academy [1]. **b** Page from the same volume containing M. Petit’s treatise “Pourquoi les enfants ne voient pas clair en venant au monde,” in which he presents early anatomical and physiological insights into the causes of poor visual clarity in newborns [1]

In his writing and correspondence, Pourfour du Petit stressed the importance of the size and shape of the surgical incision, the necessity of minimizing mechanical trauma, and the value of meticulous postoperative care. These principles, disseminated through academic societies and private communication with other surgeons, helped elevate operative standards in European ophthalmology [17, 22, 23] (Figs. 4 and 5).

#### Empirical exploration of refraction in the eye

Aligned with the Enlightenment’s drive to integrate physics and biology, Pourfour du Petit applied principles of optics to better understand the refractive indices of the cornea, aqueous humor, crystalline lens, and vitreous humor. He recognized that, with age, the lens underwent changes, becoming less elastic and thereby reducing accommodative capacity. While later scientists such as Hermann von Helmholtz would develop more sophisticated devices to examine accommodation and refractive errors, Pourfour du Petit’s early quantitative contributions provided a foundation for these investigations [34].

## Legacy and influence

### Interactions with contemporary researchers

During the early eighteenth century, France produced a range of leading anatomists and physicians, some of whom formed close professional ties with Pourfour du Petit. Yet, confusion in historical records occasionally arises because he shared part of his name with the renowned surgeon Jean Louis Petit (1674–1750). To distinguish them, contemporaries referred to François Pourfour du Petit as “Petit le Médecin” and to Jean Louis Petit as “Petit le Chirurgien.”

### Prelude to horner syndrome clarification

Some historians of neuro-ophthalmology place Pourfour du Petit at the intellectual origin of Horner syndrome, even if the complete clinical triad was later delineated by Claude Bernard and Johann Friedrich Horner (1831–1886). His experiments and published letters, which described ocular changes due to manipulations of the cervical sympathetic chain, laid the groundwork for comprehending the complex interplay of the sympathetic pathways. The final, definitive recognition of ptosis, miosis, and anhidrosis as a syndromic triad drew significantly from the foundation he had established [7, 22, 24].

### Reasons for historical overshadowing

Despite his achievements, Pourfour du Petit remained relatively obscure outside French scientific circles for much of the eighteenth and nineteenth centuries. Several factors contributed to this neglect:

1. Limited dissemination of texts: His major writings-particularly *Lettres d’un Médecin des Hôpitaux du Roy*-were printed in small runs. Few copies survived, which hampered broader recognition [16].
2. Name confusion: As noted, he shared a surname and partial field interests with Jean Louis Petit, leading to bibliographical inaccuracies [7, 8].

In recent decades, archival research and digital access have brought his works back into scholarly focus. Researchers reevaluating the evolution of neurological concepts find his systematic experimental contributions to the study of decussation, as well as the correction of earlier misconceptions about the cranial origins of the sympathetic trunk, to be landmarks in the annals of medical progress [22, 26, 32].

## Conclusion

The life and career of François Pourfour du Petit (1664–1741) intersected an era when battlefield surgery, university-based research, and academic societies converged to foster a new form of medical science, one in which theories were tested rather than merely proposed. The specialized intersection of systematic experimentation, precise dissection, and real-world clinical experience defined Pourfour du Petit’s contributions, ensuring their resonance in the subsequent development of neuroscience and ophthalmology. Although overshadowed for a time, his name has reemerged in contemporary historical evaluations of Enlightenment medicine as a symbol of rigorous, evidence-based progress.

Through a more widespread acknowledgment of his work-made easier by ongoing archival research, bibliographic clarifications, and historical reprints-the medical community can appreciate how this 18th-century figure helped pave the way for modern understandings of neuroanatomy and ophthalmic surgery. History is a testament to the enduring power of careful empirical study, underscoring the Enlightenment ideal that verifiable experiment, not speculation, is the ultimate arbiter of scientific truth.

## Data Availability

No datasets were generated or analysed during the current study.
